# Ovarian stimulation with follitropin delta for *in vitro* fertilization: a multicentre, randomized, assessor-blind comparison with follitropin alfa using conventional dosing regimens (ADAPT-1 trial)

**DOI:** 10.1093/humrep/deaf119

**Published:** 2025-07-09

**Authors:** Andrea Bernabeu, Philipp Zajc, Marta García Sánchez, Rina Agrawal, Enrico Papaleo, Stefan Jirecek, Signe Møgelmose, Ida Engberg Jepsen, Rita Lobo

**Affiliations:** Instituto Bernabeu Alicante, Albufereta, Alicante, Spain; Babywunsch Klinik Dr Zajc, Wals-Siezenheim, Austria; Quirónsalud Málaga Hospital, Málaga, Spain; IVI Málaga, Málaga, Spain; Reproductive Medicine, University Hospitals Coventry and Warwickshire NHS Trust, Coventry, UK; Reproductive Sciences Unit, San Raffaele Hospital, Milan, Italy; Kinderwunschzentrum Doebling, Vienna, Austria; Global Biometrics, Ferring Pharmaceuticals, Kastrup, Denmark; Global Research and Medical, Ferring Pharmaceuticals, Kastrup, Denmark; Global Research and Medical, Ferring Pharmaceuticals, Kastrup, Denmark

**Keywords:** follitropin delta, FE 999049, gonadotropins, normo-responders, rFSH, ovarian stimulation, GnRH antagonist protocol, RCT, IVF, conventional dosing regimens

## Abstract

**STUDY QUESTION:**

How do ovarian responses using conventional dosing for follitropin delta 15 µg/day compare with follitropin alfa 225 IU/day in women undergoing ovarian stimulation?

**SUMMARY ANSWER:**

The ADAPT-1 trial demonstrates similar ovarian responses with follitropin delta 15 µg/day and follitropin alfa 225 IU/day starting doses in a conventional dosing regimen.

**WHAT IS KNOWN ALREADY:**

Follitropin delta, a recombinant FSH (rFSH), is currently approved for ovarian stimulation using an individualized fixed daily dose based on serum anti-Müllerian hormone (AMH) and bodyweight (maximum 12 µg/day for first cycle and 24 µg/day in subsequent cycles). Other rFSHs, such as follitropin alfa, conventionally apply a starting dose of 150–225 IU, fixed for the initial days of stimulation, after which dose adjustments can be made (maximum 450 IU/day). Ovarian stimulation with follitropin delta 10 µg/day provides a similar ovarian response to follitropin alfa 150 IU/day for serum concentration and number of follicles ≥12 mm.

**STUDY DESIGN, SIZE, DURATION:**

ADAPT-1 was a randomized, accessor-blinded, multicentre trial comparing efficacy and safety of a starting dose of follitropin delta 15 µg/day with follitropin alfa 225 IU/day in conventional dosing regimens. The primary endpoint was the number of oocytes retrieved; mean difference between treatment groups was estimated using a negative binomial regression model (treatment and serum AMH level as factors). During the follow-up period, clinical pregnancies resulting from the first fresh/frozen transfers within 3 months of the start of stimulation, and ovarian hyperstimulation syndrome (OHSS) rates were assessed.

**PARTICIPANTS/MATERIALS, SETTING, METHODS:**

Participants, 18–40 years, undergoing IVF/ICSI could enrol at specialist reproductive clinics in Austria, France, Italy, Spain, and the United Kingdom for ovarian stimulation if they had no contraindications for treatment with a starting gonadotropin dose of 225 IU/day. Patients could enrol if they reported infertility for at least 1 year if ≤37 years and at least 6 months for those >37 years, and regular menstrual cycles (21–35 days). All cycles used a GnRH antagonist protocol.

**MAIN RESULTS AND THE ROLE OF CHANCE:**

Between 1 August 2022 and 16 April 2024, 300 of 337 screened patients were randomized to, and received, follitropin delta (n = 200) or follitropin alfa (n = 100). The two treatment groups were comparable in terms of demographics, baseline characteristics, and duration of infertility. The mean duration of treatment was ∼9 days in both groups. The mean total dose of follitropin delta was 143.7 ± 33.6 µg and 154.3 ± 23.1 µg (2105 ± 315 IU) for follitropin alfa. Three-quarters (226/300) used an human Chorionic Gonadotropin trigger for final follicular maturation. A mean of 9.9 oocytes was retrieved for both groups (estimated difference: 0.0 oocytes; 95% CI −1.3, 1.2). The category of 8–14 oocytes retrieved was the most common ovarian response (follitropin delta: 45.5%; follitropin alfa: 50.0%). Clinical pregnancy rates were comparable (31.6% and 31.0%; estimated difference 0.6 (95% CI −10.6, 11.8)). Early OHSS (≤9 days after triggering) occurred in 2.5% and 3.0%, and all cases were Grade 3 (moderate) or lower. No participant had the stimulation cycle cancelled due to excessive ovarian response.

**LIMITATIONS, REASONS FOR CAUTION:**

Only pregnancies from the first fresh or cryopreserved transfer within 3 months of oocyte retrieval were recorded. Cumulative pregnancy rates after the first transfer were not followed up. All analyses are of descriptive nature and no formal hypothesis testing or multiplicity adjustment was applied.

**WIDER IMPLICATIONS OF THE FINDINGS:**

Treatment groups had similar ovarian responses, supporting equivalence for starting doses follitropin delta 15 µg and follitropin alfa 225 IU, with low rates of early OHSS. Ovarian stimulation cycles with follitropin delta in µg can be planned and adjusted, leveraging the established IU dose equivalence to follitropin alfa.

**STUDY FUNDING/COMPETING INTEREST(S):**

This trial was funded by Ferring Pharmaceuticals A/S, Kastrup, Denmark. Medical writing support for manuscript development was provided by Celia J. Parkyn, PhD, and was funded by Ferring Pharmaceuticals A/S, Kastrup, Denmark. P.Z. and R.A. have none. A.B. has received grants from Gedeon Richter, IBSA, GP Pharm & Seid, Miguel Hernandez University, Centre for Technological Development and Innovation, Valencian Innovation Agency, Regional Secretary for Industry, Trade and Consumption, and the Government Ministry of Industry and Tourism; payments from University Complutense of Madrid, Ferring Pharmaceuticals, Fentypharm, Miguel Hernandez University, Gedeon Richter, & Vall d’Hebron Hospital; travel support from Ferring Pharmaceuticals, Fentypharm, Gedeon Richter, International ESHRE Congress Organising Committee, Spanish Society of Gynaecology and Obstetrics, CROG Congress Organising Committee, & Vall d’Hebron Hospital; has a patent pending for Sperm plate (U202431456); and is Chair of the Organising Committee of the Infertility and Sterility Section of the Spanish Society of Gynaecologist (non-paid role) and equipment from Obstetrics; & equipment from Cook Medical. J.S. has received consulting fees from Ferring Pharmaceuticals. E.P. has received grants from Merck, Ferring, Theramex, Gedeon Richter, IBSA, and Organon; payments from Merck, Ferring, IBSA, and Organon; travel support from Merck, IBSA, Organon, Theramex, and Ferring; and participated in a Safety Monitoring Board or Advisory Board for Merck. M.G.S. has received patient medication from Ferring; payments from Ferring, Merck for educational events; and travel support from Ferring, Merck, Theramex, and IBSA. R.L. and S.M. are employees of Ferring Pharmaceuticals. I.E.J. was an employee of Ferring Pharmaceuticals at the time of the trial conduct.

**TRIAL REGISTRATION NUMBER:**

clinicaltrials.gov NCT05263388; Eudract 2021-001785-38

**TRIAL REGISTRATION DATE:**

16 November 2021 (clinicaltrials.gov); 5 August 2021 (Eudract)

**DATE OF FIRST PATIENT’S ENROLMENT:**

1 August 2022

## Introduction

Regulatory approval for follitropin delta, a recombinant FSH (rFSH) for ovarian stimulation, has been granted based on global registrational trials comprised of a broad range of women from countries across different continents, with different ethnicities, body weights, and anti-Müllerian hormone (AMH) levels (Nyboe Andersen *et al.*, 2017; Ishihara *et al.*, 2021; Qiao *et al.*, 2021). These trials used an individualized fixed daily dose of follitropin delta based on serum AMH and body weight. The approved maximum daily dose of follitropin delta is 12 µg for the first treatment cycle and 24 µg in subsequent treatment cycles. Other approved rFSH preparations, such as follitropin alfa and follitropin beta, apply a conventional dosing approach with a starting dose of 150–225 IU in women with a predicted normal ovarian response, fixed for the initial 4–6 days of stimulation, followed by the possibility of subsequent dose adjustments, with a maximum daily dose of 450 IU (The ESHRE Guideline Group on Ovarian Stimulation *et al.*, 2020). In clinical practice, conventional dosing of rFSH is typically individualized based on the patient’s clinical characteristics, such as markers of ovarian reserve, age, BMI, risk of ovarian hyperstimulation syndrome (OHSS), presence of polycystic ovaries and, if applicable, previous ovarian response to ovarian stimulation (The ESHRE Guideline Group on Ovarian Stimulation *et al.*, 2020; Fatemi *et al.*, 2021).

Previous analysis of over 1500 patients participating in clinical trials has established that a daily dose of follitropin delta 10 µg provides a similar response to follitropin alfa 150 IU/day for serum rFSH concentration, with good numbers of follicles ≥12 mm at the end of stimulation and the number of oocytes retrieved across trial populations of women undergoing ovarian stimulation for IVF or intracytoplasmic sperm injection (ICSI) (Arce *et al.*, 2020). Using these results, the dose of follitropin delta 15 µg can be extrapolated to be equivalent to follitropin alfa 225 IU; however, clinical data are required to test whether these higher doses are indeed equivalent. The objective of this trial was to compare a starting dose of follitropin delta 15 µg/day with follitropin alfa 225 IU/day in conventional dosing regimens with respect to ovarian response in infertile women undergoing ovarian stimulation for whom a starting dose of rFSH 225 IU/day would be suitable.

## Materials and methods

### Trial design

The ADAPT-1 trial was a randomized, controlled, open-label, accessor-blinded, multicentre trial comparing the efficacy and safety of starting dose follitropin delta 15 µg/day with follitropin alfa 225 IU/day in conventional dosing regimens in patients aged 18–40 years undergoing ovarian stimulation cycle for IVF/ICSI.

The ADAPT-1 trial was conducted in compliance with the International Council for Harmonisation guideline on Good Clinical Practice (GCP). The study protocol and all relevant materials used for the trial were approved by the Institution Ethics Committees for each trial site. Participants provided informed written consent. All investigators were required to have completed GCP training and accreditation before the start of the trial, as well as training on the trial protocol.

### Trial participants

Participants, 18–40 years, could enrol at specialist reproductive health clinics in Austria, France, Italy, Spain, the United Kingdom and were eligible for IVF or ICSI due to infertility. The main inclusion criteria for trial participation were infertility for at least 1 year for participants ≤37 years and at least 6 months for those >37 years, good physical and mental health judged by the investigator, presence of two ovaries without significant abnormalities, tubal infertility, unexplained infertility, endometriosis stage I/II or partners with male factor infertility, and regular menstrual cycles (21–35 days). Women with known endometriosis stages III–IV, endometrioma >3 cm or known reason contraindicating the use of gonadotropins 225 IU/day (such as previous episode of OHSS, exuberant ovarian response to gonadotropins, polycystic ovarian syndrome, enlarged ovaries, or ovarian cyst), or if judged by the investigator as not suitable for ovarian stimulation with gonadotropin 225 IU/day were not permitted to enrol. The full list of inclusion/exclusion criteria is provided in Supplementary Table S1.

### Trial endpoints

The primary endpoint was the number of oocytes retrieved, and secondary endpoints included ovarian response (follicular development, endocrine profile, and oocyte quality). Follicular development (number and size of follicles) and endocrine profile (serum oestradiol and progesterone) were assessed at the end of stimulation. Total gonadotropin (rFSH) exposure (mean daily dose and total dose) and duration of treatment were also evaluated. There was a follow-up period to collect secondary endpoint information from the participant’s first fresh (Day 5) or frozen Day 5/6 blastocyst transfer visit if conducted within 3 months of the start of stimulation. Fertilization rate, the number and quality (based on the Gardner & Schoolcraft blastocyst scoring system; Gardner and Schoolcraft, 1999) of transferred blastocysts (Day 5/6), and clinical pregnancy at 5–6 weeks after transfer were also assessed during the follow-up period. For participants who underwent a transfer in the fresh cycle, assessment of late OHSS (onset >9 days after triggering) took place during the follow-up period.

### Randomization and treatment

Participants were screened within 90 days prior to randomization. On days 2–3 of their menstrual cycle, participants were randomized in 2:1 ratio to follitropin delta 15 µg or follitropin alfa 225 IU following a GnRH antagonist protocol. Randomization was stratified by trial site using a computer-generated randomization list operated via an interactive response technology system (Endpoint Clinical Inc., Wakefield, United States). Based on an established dose equivalence factor, the ovarian response with the starting dose of follitropin delta 15 µg/day was expected to be comparable to follitropin alfa 225 IU/day (16.5 µg/day) (Arce *et al.*, 2020). The dose of each rFSH was fixed for the first 4 days, and thereafter dose adjustments could be implemented on the day of starting the GnRH antagonist (stimulation Day 5 or 6) or later and could occur no more frequently than once every other day. At each dose adjustment, the daily dose could be increased or decreased by 5 µg (follitropin delta) or 75 IU (5.5 µg; follitropin alfa) based on the participant’s response to follitropin delta and follitropin alfa, respectively (minimum daily dose, 5 µg/75 IU and maximum dose, 20 µg/300 IU).

The two trial medications have different-looking injection pens (the commercially available pens were used in the trial). Blinding would have required a double-blind, double-dummy design which would have added an extra burden on the participants. Instead, the assessor-blind design ensured that the investigators and other assessors such as embryologists were blinded to individual treatment allocation and did not have access to this information in the electronic data capture (EDC) system. The trial medication personnel, trial monitors, and the participants were aware of treatment allocation after randomization. Participants were instructed not to mention their trial treatment to the investigator and to refer any queries about trial medication to the trial medication personnel.

### Trial procedures

On stimulation Day 5 or 6, a GnRH antagonist (ganirelix acetate 0.25/day, FYREMADEL^®^, SUN Pharma, Mumbai, India) was administered to prevent a premature luteinizing hormone surge and was continued throughout the stimulation period. A single dose of human chorionic gonadotropin 250 µg (hCG; OVITRELLE^®^, Merck, Darmstadt, Germany) was administered as soon as each participant achieved the criterion for triggering of final follicular maturation (≥3 follicles with a diameter ≥17 mm; monitored by transvaginal ultrasound). Triggering could also be done when one or two follicles with a diameter ≥17 mm were observed if the investigator judged that ≥3 follicles with a diameter ≥17 mm could not be reached, and that triggering was preferred instead of cycle cancellation. If there were ≥25 follicles with a diameter ≥12 mm or the serum oestradiol was ≥5000 pg/ml (18 355 pmol/l) (local laboratory) or a freeze-all approach was intended, a GnRH agonist (triptorelin acetate 0.2 mg, GONAPEPTYL^®^, Ferring, Kasrup, Denmark) trigger was used; these criteria were used to align with previous follitropin delta clinical trials (Nyboe Andersen *et al.*, 2017; Ishihara *et al.*, 2021; Qiao *et al.*, 2021). A GnRH agonist trigger could also be used if the investigator judged that the participant was at risk of developing early OHSS (onset ≤9 days after triggering). Cycles were cancelled if the investigator judged that the triggering criterion could not be reached by stimulation Day 20 (poor ovarian response) or if there were safety concerns due to excessive ovarian response as judged by the investigator based on local practices.

Oocyte retrieval took place 36 ± 2 h after triggering of final follicular maturation. Administration of rFSH was not allowed after the participant met the triggering criteria. The oocytes could be inseminated by IVF and/or ICSI. Fertilization was assessed on Day 1 after oocyte retrieval. Blastocyst culture and Day 5 embryo-transfer were mandatory for a fresh transfer. Blastocysts that were not transferred in the fresh cycle were cryopreserved in accordance with local guidelines and/or regulations.

For participants who underwent triggering of final follicular maturation, the end-of-trial visit had to take place 9–14 days after triggering to cover the assessment of early OHSS (onset ≤9 days after triggering). If the cycle was cancelled prior to triggering, the end-of-trial assessments were performed at the last scheduled trial visit or as a separate visit within 7 days of the last visit.

#### Follow-up period transfer procedures

Depending on blastocyst availability, it was expected that subjects who had undergone triggering of final follicular maturation with hCG would undergo transfer on Day 5 after oocyte retrieval and that subjects who had undergone triggering of final follicular maturation with GnRH agonist would undergo transfer in a frozen cycle using blastocysts cryopreserved on Day 5 or 6 after oocyte retrieval. Unless unavailable, only good-quality blastocysts, defined as Grade 3BB (Gardner and Schoolcraft, 1999), or higher, were permitted to be transferred within the trial. If a participant had no good-quality blastocyst available, transfer of a non-good-quality blastocyst or morula was allowed. The number of transferred blastocysts in the first fresh or frozen cycle was based on the participant’s wishes and the investigator’s recommendation and in accordance with local guidelines and/or regulations. All participants with a blastocyst or non-blastocyst transfer were followed to clinical pregnancy. Luteal phase support in fresh and frozen cycles as well as potential other medicinal products for programming of frozen cycles was in accordance with the site’s clinical practice. Clinical pregnancy from the first transfer cycle was assessed by transvaginal ultrasound 5–6 weeks after transfer, and the number of gestational sacs was recorded.

### Bioanalysis

Blood samples were collected at stimulation Day 1 for measurement of serum AMH, oestradiol, and progesterone as well as at end-of-stimulation for measurement of serum oestradiol and progesterone.

### Data quality assurance and trial monitoring

The trial EDC system was provided by an independent third-party contract research organization (Viedoc Technologies AB, Uppsala, Sweden). Any errors in the EDC system were corrected electronically and tracked by an audit trail detailing the date and time and reason of the correction and the name of the person making the correction. Trial monitoring, adherence to the trial protocol, general quality, and data consistency checks were regularly conducted by the sponsor throughout the trial and the post-trial follow-up period in accordance with GCP guidelines via on-site visits, remote visits, telephone calls, and checking the accuracy of EDC entries compared to source data.

### Statistical analysis

The primary endpoint, number of oocytes retrieved, was compared between follitropin delta and follitropin alfa using a negative binomial regression model with treatment and serum AMH level at stimulation Day 1 (AMH <15 pmol/l, AMH ≥15 pmol/l, or missing) as factors. The absolute mean treatment difference in number of oocytes retrieved and the associated 95% CI were derived from the model estimates using the delta method. The primary analysis was conducted for the full analysis set (defined as all randomized participants exposed to the trial drug and analysed according to the planned [randomized] treatment) and the per-protocol analysis set. The treatment difference was also investigated for subgroups defined by AMH level at stimulation Day 1 (i.e. <15 pmol/l, AMH ≥15 pmol/l or missing) using the same methods as above for each subgroup separately. No formal hypothesis testing or multiplicity adjustment was conducted. Secondary ovarian response endpoints were compared between treatments using the same negative binomial regression model as for the primary endpoint. Missing observations for oocytes retrieved, fertilized oocytes, and number of blastocysts were replaced with zero (i.e. worst-case imputation technique). Implantation and clinical pregnancy rates were compared between treatments using a negative binomial regression model and a logistic regression model, respectively, both with treatment and age group (<35, 35–37, or 38–40 years) as factors. The estimated means, difference in means, and associated 95% CIs were presented based on the delta method. The analysis of implantation rates was based only on subjects with a transfer and the number of transferred blastocysts was accounted for by introducing an offset in the model. In the analysis of pregnancy rates, participants with missing data (i.e. participants with no transfer) were regarded as non-pregnant.

Serum concentrations of oestradiol and progesterone at end-of-stimulation were compared between treatments using analysis of covariance (ANCOVA) models with treatment and AMH level at stimulation Day 1 (AMH <15 pmol/l, AMH ≥15 pmol/l, or missing) as factors and with the serum concentration (log-transformed) at stimulation Day 1 as a covariate. Multiplicative models were used, i.e. the serum concentrations were log-transformed before analysis. The results of the analyses were back-transformed and presented as estimated geometric means and mean treatment ratios with associated 95% CIs. Values below the lower limit of quantification (LLOQ) were estimated with LLOQ/2. Potential values above the upper limit of quantification (ULOQ) were included as ULOQ.

The number of stimulation days was compared between treatments using an analysis of variance model with treatment and AMH level at stimulation Day 1 (AMH <15 pmol/l, AMH ≥15 pmol/l, or missing) as factors.

#### Sample size

We calculated the required sample size using data from a previous trial (MERIT), in which 347 participants aged 22–37 years treated with follitropin alfa at a starting dose of 225 IU had a mean of 11.8 ± 5.7 oocytes retrieved (Nyboe *et al.*, 2006). Assuming that the SD for number of oocytes retrieved in our trial would be 6.0, a total sample size of 300 with a 2:1 randomization would result in a 2-sided 95% CI ranging from −1.44 to +1.44 oocytes for the observed difference. This level of precision was considered sufficient and more precise than the equivalence limits of ±3 oocytes that had been applied in previous trials comparing biosimilar FSH compounds (Rettenbacher *et al.*, 2015; Barakhoeva *et al.*, 2019; Hu *et al.*, 2020).

## Results

### Disposition and baseline characteristics

**Figure 1.**
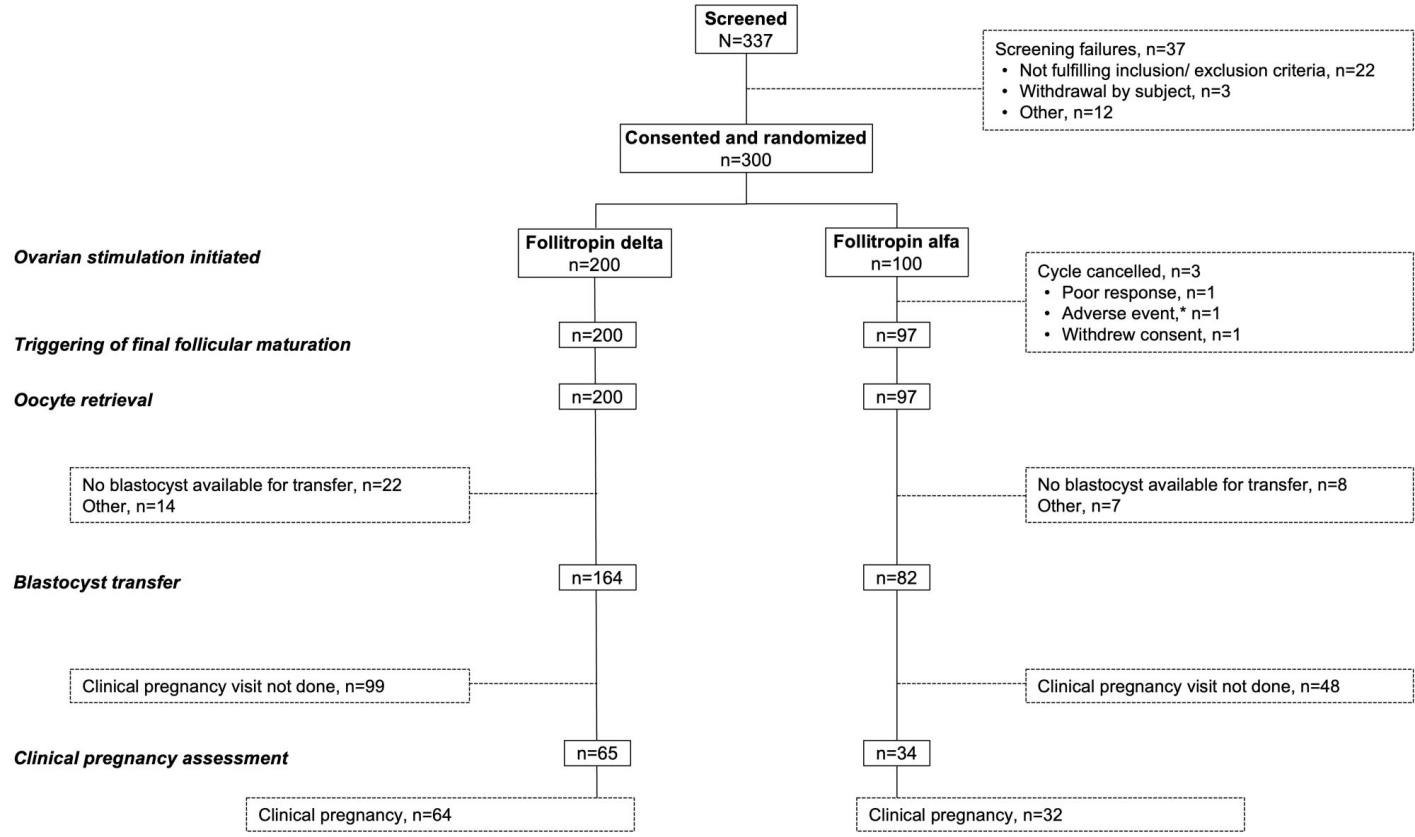
**ADAPT-1 trial participant flow.** *Cycle cancelled due to a pre-treatment adverse event that was only found out about after treatment had started.

Between 1 August 2022 and 16 April 2024, a total of 337 patients were screened and 300 were randomized to, and correctly received, follitropin delta (n = 200) or follitropin alfa (n = 100) (full analysis set; Fig. 1). The full analysis set was identical to the intention-to-treat analysis set. Of the 37 screening failures, 22 did not meet the inclusion/exclusion criteria, and 15 were not randomized due to various reasons (patient withdrawal, n = 3; >90 days lapsed since screening, n = 3; lost contact, n = 3; trial closed prior to randomization, n = 2, other reasons, n = 4). There were 10 participants who had protocol violations and were not included in the per-protocol analysis set (follitropin delta group, n = 7; follitropin alfa group, n = 3). There were 17 trial sites across five European countries (Austria, France, Italy, Spain, and the United Kingdom). Ten participants did not complete the main trial (follitropin delta, n = 4; follitropin alfa, n = 6). Reasons for trial discontinuation included: an adverse event (follitropin alfa, n = 2); withdrawal of consent (follitropin delta, n = 1; follitropin alfa, n = 1); started a second ovarian stimulation cycle during the luteal phase (follitropin delta, n = 1); cycle cancellation due to poor ovarian response (follitropin alfa, n = 1); did not attend end-of-trial visit (follitropin delta, n = 1; follitropin alfa, n = 2); and lost to follow-up after oocyte retrieval (follitropin delta, n = 1). During the trial follow-up period, 164 (82.0%) and 82 (82.0%) participants underwent a blastocyst transfer in the follitropin delta and follitropin alfa groups, respectively.

The two treatment groups were comparable in terms of demographics, baseline characteristics, and duration of infertility (Table 1). There were some group differences in primary reason for infertility with a slightly higher proportion of participants in the follitropin delta group having unexplained infertility compared with the follitropin alfa group (41.5% and 33.0%, respectively) and slightly lower proportions with tubal (11.5% and 14.0%) or male factor (42.5% and 49.0%) infertility. Overall, the serum AMH concentration on stimulation Day 1 was similar between treatment groups and 57.0% of the trial population had a serum AMH <15 pmol/l (seven participants had missing data for baseline serum AMH). Serum oestradiol and progesterone levels on Day 1 of stimulation were also similar between the two treatment groups.

**Table 1.** Demographics and baseline characteristics (full analysis set).

	Follitropin delta Starting dose 15 µg/day, n = 200	Follitropin alfa Starting dose 225 IU/day, n = 100
Age, years	34.5 ± 3.8	34.5 ± 3.4
Age category		
<35 years	83 (41.5)	43 (43.0)
35–37 years	74 (37.0)	39 (39.0)
38–40 years	43 (21.5)	18 (18.0)
Race		
American Indian or Alaska Native	1 (0.5)	1 (1.0)
Asian	7 (3.5)	0
Black or African American	1 (0.5)	1 (1.0)
White	191 (95.5)	98 (98.0)
Ethnicity		
Hispanic or Latino	27 (13.5)	20 (20.0)
Not Hispanic or Latino	173 (86.5)	80 (80.0)
Bodyweight, kg	66.4 ± 13.0	67.5 ± 11.3
BMI, kg/m^2^	24.65 ± 4.61	24.75 ± 3.96
Primary reason for infertility		
Unexplained infertility	83 (41.5)	33 (33.0)
Tubal infertility	23 (11.5)	14 (14.0)
Male factor	85 (42.5)	49 (49.0)
Endometriosis stage I/II	9 (4.5)	4 (4.0)
Primary infertility	134 (67.0)	58 (58.0)
Duration of infertility, months	36.7 ± 28.6	35.8 ± 24.8
Antral follicle count	13.3 ± 5.5	13.8 ± 5.9
Endocrine parameters on stimulation Day 1		
AMH, pmol/l	14.5 ± 7.7 [n = 197]	15.2 ± 11.0 [n = 96]
AMH category		
<15 pmol/l	112 (56.9)	59 (61.5)
≥15 pmol/l	85 (43.1)	37 (38.5)
Missing	3 (1.5)	4 (4.0)
Oestradiol, pmol/l	136.0 (106.0–180.0) [n = 193]	125.0 (103.0–169.0) [n = 96]
Progesterone, nmol/l	0.6 (0.6–1.0) [n = 195]	0.6 (0.6–1.0) [n = 97]

Data are n (%), mean±SD or median (interquartile range).

### Use of non-investigational drugs

All 300 participants who started ovarian stimulation were exposed to GnRH antagonist (ganirelix acetate 0.25 mg/day). The mean duration of GnRH antagonist exposure was similar in the treatment groups (follitropin delta, 5.1 ± 1.7 days; follitropin alfa, 5.0 ± 1.4 days).

The most frequently used concomitant medications (defined using the WHO Drug Reference List) during ovarian stimulation were anaesthetics (98.0%), sex hormones, and modulators of the genital system predominantly used to support implantation (63.7%), analgesics (52.3%), and anti-anaemic preparations (52.3%); concomitant medications were mostly prescribed as part of the oocyte retrieval regimen as per local practice, and their use was comparable between the treatment groups.

#### Triggering of final follicular maturation

Of the 300 participants who started ovarian stimulation, 200 (100%) and 97 (97%) participants in the follitropin delta and the follitropin alfa groups, respectively, underwent triggering of final follicular maturation. The reasons for cycle cancellation for the three participants in the follitropin alfa group who did not undergo triggering were poor ovarian response (n = 1), cycle cancellation due to an adverse event prior to starting ovarian stimulation (n = 1; the participant had an asthma exacerbation that required pneumological follow-up) and participant withdrawal of consent (n = 1). Approximately three-quarters (226/300 [75.3%]) of participants used an hCG trigger and 23.7% (71/300) used a GnRH agonist trigger (Table 2).

**Table 2.** Gonadotropin exposure, ovarian response, fertilization, and blastocyst transfer (full analysis set).

	Follitropin delta Starting dose 15 µg/day, n = 200	Follitropin alfa Starting dose 225 IU/day, n = 100	**Difference (95% CI)** ^a^
Exposure to trial drug			
Duration, days^b^	9.2	9.0	0.1 (−0.2, 0.5)
Daily dose, µg	15.6 ± 1.4	17.1 ± 1.2	–
Total dose, µg	143.7 ± 33.6	154.3 ± 23.1	–
Total dose, IU	–	2105 ± 315	–
Participants with investigator-requested gonadotropin dose adjustments	66 (33.3)	28 (28.0)	–
Triggering of final follicular maturation^c^			
hCG	152 (76.0)	74 (74.0)	–
GnRH agonist^d^	48 (24.0)	23 (23.0)	–
None	0	3 (3.0)	–
Ovarian response			
Size of follicles ≥10 mm at end of stimulation, mm	15.9 ± 1.4 [n = 200]	16.0 ± 1.3 [n = 99]	_
Follicles ≥10 mm^e^	12.9	12.7	0.2 (−1.0; 1.5)
Follicles ≥12 mm^e^	11.3	11.3	0.0 (−1.2, 1.2)
Follicles ≥15 mm^e^	8.1	8.4	−0.3 (−1.3, 0.7)
Follicles ≥17 mm^e^	6.0	5.9	0.1 (−0.7, 0.9)
Endocrine profile at end of stimulation^f^			
Oestradiol, pmol/l	5462.8 [n = 192]	6127.2 [n = 92]	Ratio 0.9 (0.8, 1.0)
Progesterone, nmol/l	2.6 [n = 194]	2.4 [n = 93]	Ratio 1.1 (0.9, 1.2)
Oocytes, fertilization, and blastocysts			
Oocytes retrieved^e^	9.9	9.9	0.0 (−1.3, 1.2)
MII oocytes^g^	7.2 ± 4.57 [n = 135]	8.1 ± 5.12 [n = 73]	_
MII oocytes^g^/oocytes retrieved	72.2 ± 17.96 [n = 135]	75.1 ± 18.98 [n = 73]	_
Fertilized oocytes^e^	5.5	5.4	0.1 (−0.8, 1.0)
Fertilization rate relative to oocytes retrieved^e^	55.1% [n = 198]	54.3% [n = 96]	0.8 (−4.8, 6.4)
Total blastocysts on Day 5/6^e^	3.2	3.1	0.2 (−0.4, 0.8)
Good-quality^e^^,^^h^	2.3	2.1	0.1 (−0.4, 0.6)
Total blastocysts on Day 5	2.5 ± 2.48	2.3 ± 2.15	_
Total blastocysts on Day 6	0.8 ± 1.48	0.8 ± 1.56	_

Data are mean±SD, n (%), adjusted mean estimates (for ovarian response parameters) or geometric mean (for endocrine parameters).

a Differences and CIs derived using the delta method.

b ANOVA model with treatment and AMH group as factors.

c Cycle cancellation was stipulated by the protocol if the investigator judging that the triggering criterion could not be reached by Day 20. Triggering criterion were observation of ≥3 follicles with a diameter ≥17 mm (i.e. on the day or the day after observation). Triggering could also be done if one or two follicles with a diameter ≥17 mm were observed and the investigator judged that ≥3 follicles with a diameter ≥17 mm could not be reached, and that triggering was preferred instead of cycle cancellation.

d An GnRH agonist trigger could be used final follicular development if there were ≥25 follicles with a diameter ≥12 mm or the serum oestradiol is ≥5000 pg/ml (18 355 pmol/l) (local laboratory).

e Negative binomial regression model was based on the full analysis set with treatment and AMH group as factors.

f Multiplicative ANCOVA model with treatment and AMH group as factors and baseline serum concentration as covariate.

g MII oocytes with the presence of a polar body and two pronuclei were identified for participants who had intracytoplasmic sperm injection as the only method of fertilization.

h Good-quality blastocysts were defined as Grade 3BB or higher (Gardner and Schoolcraft, 1999).

AMH, anti-Müllerian hormone; MII, metaphase II.

### Ovarian stimulation

Gonadotropin (rFSH) dosing for ovarian stimulation is summarized in Table 2. Overall, the mean duration of treatment was similar between treatment groups (∼9 days). The mean total dose of gonadotropin used was 143.7 ± 33.6 µg for follitropin delta and 154.3 ± 23.1 µg (2105 ± 315 IU) for follitropin alfa. Approximately one-third of participants (33.0% [66/200] and 28.0% [28/100] in the follitropin delta and follitropin alfa groups, respectively) had their investigator request a dose adjustment, with the vast majority of requests being for dose increases.

### Ovarian response

#### Number of oocytes retrieved (primary endpoint)

**Figure 2.**
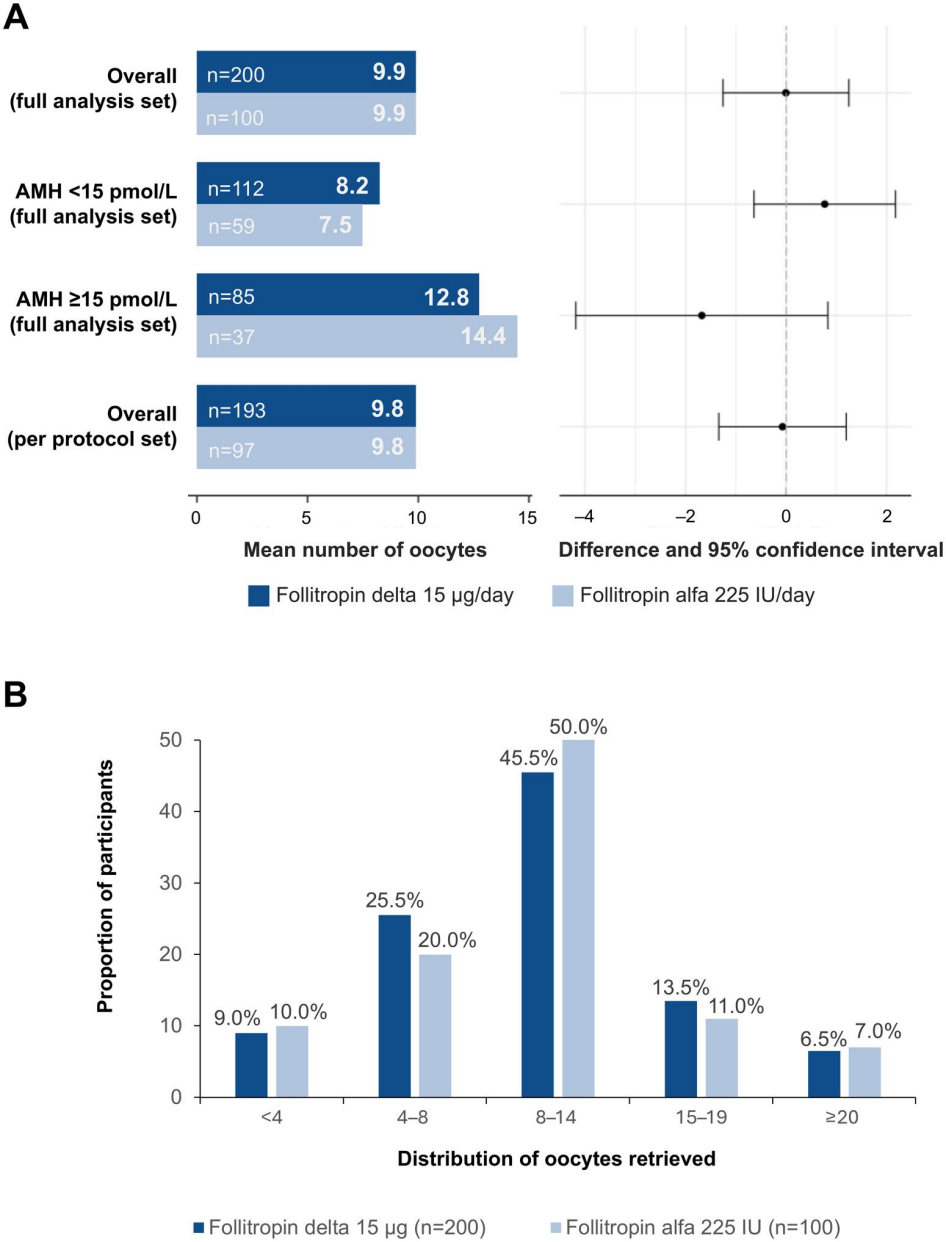
**Number of oocytes retrieved (primary endpoint; full analysis set).** (**A**) Adjusted treatment comparison for overall and AMH subgroup. Treatment comparison is adjusted for or by AMH subgroup. The mean treatment differences for AMH subgroups deviate slightly from 0 in opposite directions, but 95% CIs include 0 (zero). There were 10 participants who had protocol violations and were not included in the per-protocol analysis set (follitropin delta group, n=7; follitropin alfa group, n=3). (**B**) Distribution of oocytes retrieved. Participants with cycle cancellation due to poor ovarian response are included in the <4 oocytes group. The full analysis set comprised all randomized participants exposed to the trial drug and analysed according to the planned treatment and was identical to the intention-to-treat analysis set. AMH, anti-Müllerian hormone.

All participants with triggering of final follicular maturation underwent oocyte retrieval. The estimated mean number of oocytes retrieved was 9.9 for both treatment groups and the estimated treatment difference was 0.0 oocytes, with a 95% CI of −1.3 to 1.2 (Fig. 2A). Similar results were obtained when performing the per-protocol analysis. The mean number of oocytes retrieved was also comparable between treatment groups in each AMH subgroup. The category of 8–14 oocytes retrieved was the most common ovarian response, achieved by 45.5% and 50.0% of participants in the follitropin delta and follitropin alfa groups, respectively (Fig. 2B).

There were no cycle cancellations prior to oocyte retrieval due to excessive ovarian response in either treatment group. Zero and one participant had oocyte retrieval cancelled due to poor ovarian response in the follitropin delta and follitropin alfa groups, respectively.

##### Follicle size and endocrine parameters at end of stimulation

At the end of stimulation, the mean size of follicles ≥10 mm was 15.9 and 16.0 mm for follitropin delta and follitropin alfa, respectively. The number of follicles by minimum size was comparable between groups (Table 2).

Similarly, serum levels of oestradiol and progesterone were comparable between both treatment groups (Table 2).

#### Fertilization rate, blastocysts, implantation, and pregnancy

The fertilization rate was similar between treatment groups, as was the mean number of blastocysts and good-quality blastocysts at Day 5/6 (Table 2). Correspondingly, the implantation rate and clinical pregnancies were also similar between the two groups (Table 3).

**Table 3.** Follow-up efficacy assessments (full analysis set).

	Follitropin delta Starting dose 15 µg/day, n = 200	Follitropin alfa Starting dose 225 IU/day, n = 100	**Difference (95% CI)** ^a^
Participants with a blastocyst transfer^b^	164 (82.0)	82 (82.0)	–
Number of blastocysts transferred^c^	171	86	–
Number of gestational sacs 5–6 weeks after transfer	67	32	–
Adjusted implantation rate^d^	39.0%	36.2%	2.8 (−12.9, 18.4)
Adjusted clinical pregnancy rate per started cycle^e^	31.6%	31.0%	0.6 (−10.6, 11.8)

Data are n (%) or adjusted mean estimates.

a Differences and CIs derived using the delta method.

b Includes non-blastocyst transfers: six participants in the follitropin delta group and six participants in the follitropin alfa group had non-blastocyst transfers.

c Includes eight non-blastocyst transfers among the follitropin delta group and seven non-blastocyst transfers among the follitropin alfa group.

d Negative binomial regression model was based on the full analysis set with treatment and age group as factors and transferred blastocysts as offset.

e Logistic regression model was based on the full analysis set with treatment and age group as factors.

### Safety outcomes

The proportion of participants who experienced adverse events was similar for the follitropin delta and follitropin alfa groups (36.5% and 40.0%, respectively; Table 4). The two treatment groups were comparable with respect to frequency and type of treatment-emergent adverse events (TEAEs); the most commonly reported TEAEs were headache (11.5% and 8.0%), pelvic discomfort (3.5% and 4.0%), procedural pain (3.0% and 4.0%), and early onset OHSS (2.5% and 3.0%). All incidences of procedural pain were related to the oocyte retrieval procedure. The vast majority of TEAEs were mild in intensity. There were two serious adverse events, both cases of haemoperitoneum after oocyte retrieval in the follitropin alfa group; both were judged by the investigator as having no reasonable possibility of being caused by the trial drug and led to trial discontinuation for one participant. There were very few injection site reactions in either treatment group (follitropin delta, n = 2 [1.0%]; follitropin alfa, n = 2 [2.0%]). All participants with an adverse event were followed until recovery or the adverse event was resolved.

**Table 4.** Adverse event summary (safety analysis set).

	Follitropin delta Starting dose 15 µg/day, n = 200	Follitropin alfa Starting dose 225 IU/day, n = 100
Adverse events	73 (36.5)	40 (40.0)
Serious adverse events	0	2 (2.0)
Adverse drug reactions	39 (19.5)	22 (22.0)
Adverse events leading to discontinuation	0	1 (1.0)
Severe adverse events	0	2 (2.0)
Adverse events leading to death	0	0
Treatment emergent adverse events (≥3% in either group)		
Headache	23 (11.5)	8 (8.0)
Pelvic discomfort	7 (3.5)	4 (4.0)
Procedural pain	6 (3.0)	4 (4.0)
Any OHSS, any grade	7 (3.5)	3 (3.0)
Early OHSS (≤9 days after triggering), any grade	5 (2.5)	3 (3.0)
Early OHSS, Grade 1 or 2	4 (2.0)	2 (2.0)
Early OHSS, Grade 3	1 (0.5)	1 (1.0)
Any preventive intervention for potential early OHSS	30 (15.0)	15 (15.0)
Triggering with GnRH agonist	27 (13.5)	14 (14.0)
Administration of dopamine agonist	5 (2.5)	1 (1.0)
Cycle cancellation	0	0
Early OHSS and/or any preventive intervention	33 (16.5)	17 (17.0)
Late OHSS, (>9 days after triggering)	2 (1.0)	0
Late OHSS, Grade 3	2 (1.0)	0
Fatigue	8 (4.0)	2 (2.0)
Pelvic pain	1 (0.5)	5 (5.0)
Breast tenderness	3 (1.5)	4 (4.0)
Dysmenorrhoea	2 (1.0)	3 (3.0)
Nausea	1 (0.5)	3 (3.0)
Breast swelling	0	3 (3.0)

Data are n (%).

OHSS, ovarian hyperstimulation syndrome.

#### Ovarian hyperstimulation syndrome

Early OHSS (≤9 days after triggering) occurred in 2.5% and 3.0% for the follitropin delta and follitropin alfa groups, respectively, and all cases were Grade 3 (moderate) or lower (Golan *et al.*, 1989). Two participants developed Grade 3 early OHSS, both of whom had AMH ≥15 pmol/l and had undergone triggering with hCG. No participant had the stimulation cycle cancelled due to excessive ovarian response. Preventive interventions for early OHSS were implemented in 15.0% of participants in each treatment group (follitropin delta group, n = 30; follitropin alfa group, n = 15), the vast majority of whom were triggered with a GnRH agonist as the preventive intervention (follitropin delta group, n = 27; follitropin alfa group, n = 14). Six participants received a dopamine agonist (follitropin delta, n = 5; follitropin alfa, n = 1). Two in the follitropin delta group received both preventive interventions but one had not met the criterion of ≥20 follicles of ≥12 mm (recorded as a protocol deviation). Two participants developed late OHSS (>9 days after triggering) in the follitropin delta group. Both had AMH ≥15 pmol/l on Day 1 of stimulation, had fresh blastocyst transfers, and were later documented to have an ongoing pregnancy.

## Discussion

The ADAPT-1 trial is the first European randomized controlled trial to evaluate ovarian stimulation using a conventional dosing regimen for follitropin delta with a starting dose of 15 µg compared with 225 IU of follitropin alfa. The mean number of retrieved oocytes was 9.9 in both treatment groups. In addition, the follicular development, endocrine profile, and blastocyst development were comparable between treatment groups. The similarity of the observed ovarian response between treatment groups supports dosing equivalence for ovarian response with starting doses of follitropin delta 15 µg and follitropin alfa 225 IU when using a conventional dosing regimen for which dose adjustments may be applied depending on individual response. This study strongly supports previous findings described by Arce *et al.* (2020) on the clinical equivalence factor for follitropin delta 10 µg and follitropin alfa 150 IU (11 µg) providing a similar ovarian response evaluated by the number of oocytes retrieved across trial populations of women undergoing ovarian stimulation for IVF/ICSI. Arce *et al.* speculated about dose equivalence between follitropin delta 15 µg and follitropin alfa 225 IU.

In the ADAPT-1 trial, a lower mean total dose of gonadotropin was used for the follitropin delta group compared with the follitropin alfa group suggesting a potential difference in biopotency. The total gonadotrophin dose in micrograms was numerically lower in the follitropin delta (143.7 µg) versus follitropin alfa (154.3 µg (2104 IU; biological conversion factor 150 IU = 11 µg)), achieving similar ovarian response and clinical pregnancy outcomes, which supports Arce *et al.* (2020) who showed a similar ovarian response with follitropin delta when administered at a lower microgram weight dose compared with follitropin alfa (10 vs 11 µg). FSH activity, measured in IU, is determined *in vivo* by the rat-based Steelman-Pohley bioassay; however, equivalent dosing in IU resulted in a higher median number of follicles with follitropin delta compared with follitropin alfa in both the rat-based Steelman-Pohley bioassay and when assessed in humans (Olsson *et al.*, 2014). Additional research is required to confirm potential potency differences between rFSH preparations.

Gonadotropin dose adjustments were requested for slightly less than one-third of participants in ADAPT-1, which is less than in other studies in which ∼40% of cycles evaluated had dose adjustments (Mahony *et al.*, 2021). The slightly lower proportion of dose adjustments may reflect that the patient population in the ADAPT-1 trial was well-suited to the starting dose of follitropin delta 15 µg or follitropin alfa 225 IU.

In the ADAPT-1 trial, participants had an overall mean age of 34.5 years, a baseline mean bodyweight of 66.4 and 67.5 kg, mean antral follicle count of 13.3 and 13.8, and serum AMH mean of 14.5 and 15.2 pmol/l for the follitropin delta and follitropin alfa groups, respectively, reflecting real-world infertile patients at specialist reproductive health clinics for whom clinicians would likely treat with a starting dose of follitropin alfa 225 IU. Optimal ovarian response in terms of oocytes retrieved is normally achieved with rFSH doses ranging from 150 to 225 IU/day; the higher daily dose is normally used in women who are either predicted low or normal responders, but not those who are predicted high responders (The ESHRE Guideline Group on Ovarian Stimulation *et al.*, 2020).

In ADAPT-1, the implantation rate and clinical pregnancy rate for the first fresh or frozen Day 5/6 blastocyst transfer were comparable between the follitropin delta and follitropin alfa groups. Moreover, the clinical pregnancy rates were in the expected range for the ADAPT-1 trial population, taking into account their age, bodyweight, and serum AMH levels, and who could have undergone previous ART treatment cycles. Pregnancy rates for infertile women younger than 40 years, using their own oocytes, are expected to be ∼30% for the first cycle, decreasing to ∼20% by the fourth cycle (Smith *et al.*, 2015).

The safety profile for ovarian stimulation with follitropin delta with a starting dose of 15 µg was generally comparable with follitropin alfa 225 IU in a conventional dosing regimen. The incidence of OHSS was low (<3.5%), and preventive interventions for early OHSS were implemented in a similar proportion of participants in both treatment groups. It should be noted that in our trial the cut-off for using a GnRH agonist trigger as a preventive intervention for early OHSS (25 follicles, ≥12 mm diameter) was higher than the threshold often used in clinical practice (18–19 follicles, ≥11 mm diameter) (Papanikolaou *et al.*, 2006; Griesinger *et al.*, 2016). Nevertheless, investigators retained the discretion to use a GnRH agonist trigger below the protocol-defined threshold if the patient was deemed to be at increased risk of developing OHSS based on their clinical judgement.

The low rate of OHSS among the trial participants may be in part explained by the trial population, who had been selected as being suitable for ovarian stimulation with a starting dose of 225 IU.

Apart from the dosing regimen, the ADAPT-1 trial shared key design elements with previous randomized controlled trials for follitropin delta which used a GnRH antagonist protocol, including the ESTHER-1 (Nyboe Andersen *et al.*, 2017), GRAPE (Qiao *et al.*, 2021) and STORK (Ishihara *et al.*, 2021) trials but allowed for some elements such as oocyte retrieval cancellation, use of a GnRH agonist for triggering final follicular maturation, transfer policy (fresh or cryopreserved), or luteal phase support to be handled according to local clinical practice.

### Clinical implications

These trial results are particularly useful since they will help clinicians to better understand the follitropin delta microgram unit in comparison to other gonadotropins dosed in IU, especially for patients who have undergone previous ovarian stimulation cycles. Given the current trend for medical patient-centric approach, the findings will benefit patients who have been previously stimulated with gonadotropins dosed in IU and could benefit from treatment with follitropin delta or vice versa. As stated by Arce *et al.* (2020), it is essential to have a common reference unit for different rFSH preparations, especially when patients have a treatment history involving different protocols and different gonadotropins. Our trial establishes dose comparability between follitropin delta and follitropin alfa in ovarian responses among IVF/ICSI patients, the most relevant bioassay to test rFSH biological activity. Previously follitropin delta has been shown to provide a higher ovarian response than follitropin alfa when administered at equal units of biological activity as in the rat *in vivo* Steelman–Pohley assay (Olsson *et al.*, 2014) as well as in humans at the same microgram weight dose (Arce *et al.*, 2014). Ovarian stimulation cycles with follitropin delta in micrograms can be planned and adjusted, leveraging IU dose equivalence to follitropin alfa, or by using its individualized dosing algorithm based on bodyweight and serum AMH levels for calculating a fixed daily dose to use throughout the stimulation period.

The follitropin delta dosing algorithm continues to hold significant clinical value as it aims to an optimal oocyte yield while minimizing the risk of OHSS to optimize the live birth rate in a fresh transfer cycle. This approach, employing a fixed personalized maximum dose of up to 12 µg/day of follitropin delta, has been well established in diverse women of different ages, ethnicities, bodyweight, and ovarian reserve who enrolled in the ESTHER, STORK, and GRAPE trials (Nyboe Andersen *et al.*, 2017; Ishihara *et al.*, 2021; Qiao *et al.*, 2021), as well as post hoc analyses in high responders (Višnová *et al.*, 2021) and supporting data from a fresh transfer meta-analysis (Lobo *et al.*, 2025). In these settings, the algorithm has been shown to reduce the risk of excessive ovarian response, early OHSS, and the need for preventive interventions, particularly among normal and high responders. As such, the algorithm should not be considered obsolete but rather can be selectively applied. It remains highly relevant in first-cycle patients aiming for fresh embryo transfer and optimal oocyte yield, while a flexible, tailored dosing strategy may be preferable in experienced patients or when using alternative treatment paradigms, such as when a frozen cycle is preferred or when maximal oocyte yield is desired by the patient.

### Limitations

The ADAPT-1 trial had the limitation that the results were descriptive, and no formal hypothesis testing was performed. We also did not follow pregnant participants until live birth; however, follitropin delta and follitropin alfa have been extensively studied in randomized controlled trials for ovarian stimulation among infertile women in GnRH antagonist protocols (Nyboe Andersen *et al.*, 2017; Ishihara *et al.*, 2021; Qiao *et al.*, 2021). More recently, the efficacy and safety of individualized follitropin delta have been compared using either a GnRH agonist or an antagonist protocol in women with a serum AHM level ≤35 pmol/l (Lobo *et al.*, 2024). Participants could have a fresh or frozen transfer, but we do not know how many intended a freeze-all approach, only the number who had a GnRH agonist trigger.

## Overall conclusions

The similarity of ovarian response between treatment groups strongly supports dosing equivalence for conventionally dosed follitropin delta with a starting dose of 15 µg and follitropin alfa 225 IU. Clinical pregnancy rates of ∼31% were comparable between the treatment groups. The safety profile for follitropin delta 15 µg was generally comparable to follitropin alfa 225 IU (16.5 µg), with low OHSS rate. Understanding the dose equivalence when applying microgram dosing for follitropin delta will facilitate dosing decisions for patients who undergo successive ovarian stimulation cycles using different protocols.

## Supplementary Material

deaf119_Supplementary_Table_S1

## Data Availability

The data underlying this article will be shared on reasonable request to the corresponding author.
