# Correction to: Ovarian stimulation with follitropin delta for *in vitro* fertilization: a multicentre, randomized, assessor-blind comparison with follitropin alfa using conventional dosing regimens (ADAPT-1 trial)

**DOI:** 10.1093/humrep/deaf185

**Published:** 2025-09-15

This is a correction to Andrea Bernabeu, Philipp Zajc, Marta García Sánchez, Rina Agrawal, Enrico Papaleo, Stefan Jirecek, Signe Møgelmose, Ida Engberg Jepsen, and Rita Lobo, Ovarian stimulation with follitropin delta for *in vitro* fertilization: a multicentre, randomized, assessor-blind comparison with follitropin alfa using conventional dosing regimens (ADAPT-1 trial), Human Reproduction, Volume 40, https://doi.org/10.1093/humrep/deaf119.

The authors would like to apologise for an error in the legend for columns of the bar graph in Figure 2A of the above article. In the original article, the light blue columns were mistakenly annotated as Follitropin delta 15μg/day; the dark blue columns were mistakenly annotated as Follitropin alfa 225IU/day.

The column legends should be reversed, matching those of Figure 2B. That is, the dark blue columns represent Follitropin delta 15 μg/day and the light blue columns represent Follitropin alfa 225 IU/day.

The correct version of Figure 2 is below.

**Figure 2.**
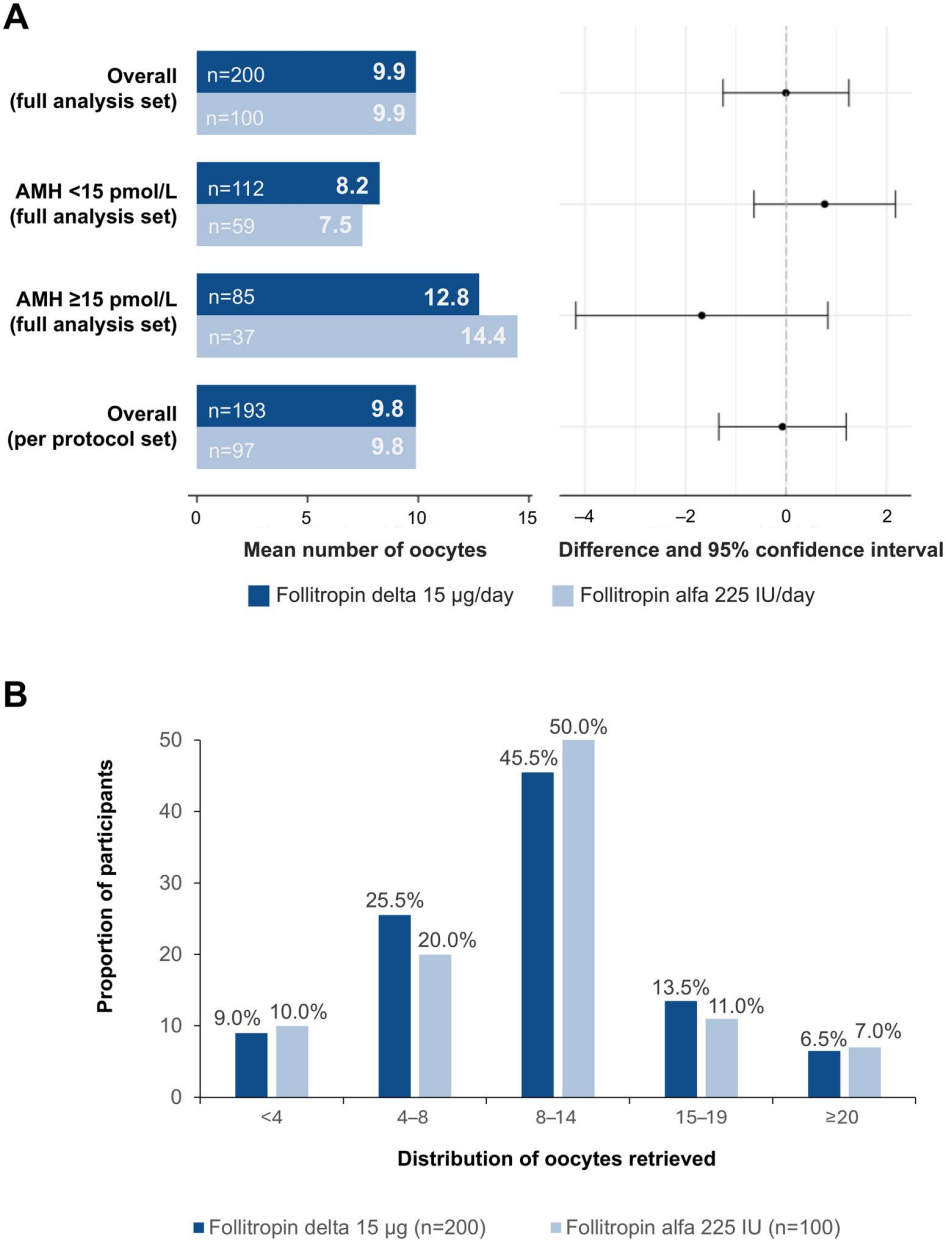
**Number of oocytes retrieved (primary endpoint; full analysis set) (A**) Adjusted treatment comparison for overall and AMH subgroup. Treatment comparison is adjusted for or by AMH subgroup. The mean treatment differences for AMH subgroups deviate slightly from 0 in opposite directions, but 95% confidence intervals include 0 (zero). There were 10 participants who had protocol violations and were not included in the per-protocol analysis set (follitropin delta group, n=7; follitropin alfa group, n=3). (**B**) Distribution of oocytes retrieved. Participants with cycle cancellation due to poor ovarian response are included in the <4 oocytes group. The full analysis set comprised all randomized participants exposed to the trial drug and analysed according to the planned treatment and was identical to the intention-to-treat analysis set. AMH, anti-Müllerian hormone.

The authors have approved this correction notice and would like to assure readers that this error does not affect any text, other analyses, or the conclusions within the article.

The electronic version of this article has been updated at https://doi.org/10.1093/humrep/deaf119.

